# Effect of a structured virtual reality simulation curriculum on laparoscopic performance in a porcine cholecystectomy model: a prospective comparative study

**DOI:** 10.1007/s00464-026-12755-z

**Published:** 2026-04-01

**Authors:** Moritz B. Sparn, Dimitrios Chatziisaak, Bruno Schmied, Walter Brunner, Martina Vitz, Lennard Ströse, Dieter Hahnloser, Stephan Bischofberger

**Affiliations:** 1 Department of Surgery, HOCH Kantonsspital St. Gallen, CH-9007 St. Gallen, Switzerland; 2 Department of Visceral Surgery, University Hospital Lausanne, Rue du Bugnon 46, CH-1011 Lausanne, Switzerland; 3 Department of Transplantation, Guy’s & St Thomas’s NHS Foundation Hospitals NHS Trust, London, UK

**Keywords:** Virtual reality simulation training, Simulation, Laparoscopic surgery training, Curriculum development

## Abstract

**Background:**

Virtual reality simulation training (VRST) has been shown to improve laparoscopic skills; however, evidence for structured simulation curricula remains limited. This study aimed to prospectively assess the effect of a structured VRST curriculum on laparoscopic performance in a porcine cholecystectomy model in novice surgeons.

**Methods:**

In this single-blinded prospective study, novice surgical trainees (< 3 years of clinical experience) attending a basic laparoscopic course were allocated to either a structured, proficiency-based VRST curriculum or standard deliberate practice on the same simulator. Laparoscopic performance was assessed during a porcine laparoscopic cholecystectomy (Lap-C) by blinded raters using the Global Operative Assessment of Laparoscopic Skills (GOALS) and a numerical rating scale (NRS).

**Results:**

Seventy-one participants were analyzed (19 intervention, 52 control). Trainees completing the structured VRST achieved significantly higher total GOALS scores compared with controls (median 17.6 vs. 14.0; *p* = 0.013). All GOALS subdomains -including depth perception, bimanual dexterity, efficiency, tissue handling, and autonomy—were significantly improved in the intervention group. Subjective performance ratings were also higher following structured VRST (median NRS 6.7 vs. 4.8; *p* = 0.036). Inter-rater reliability for the GOALS score was good (ICC = 0.80).

**Conclusion:**

Participation in a structured VR simulation curriculum improved laparoscopic performance during a standardized porcine cholecystectomy model post-test compared with deliberate practice. Integration of structured VRST curricula into surgical training programs may enhance early technical skill acquisition and procedural proficiency.

**Supplementary Information:**

The online version contains supplementary material available at https://doi.org/10.1007/s00464-026-12755-z.

Laparoscopic cholecystectomy (Lap-C) currently is the gold standard in gallbladder surgery and has fundamentally changed surgical practice over the past decades [1–4]. The technical challenges inherent to laparoscopic surgery mandate adequate training before performing Lap-C [5]. Recent publications suggest similar problems with the increasing use of robotic platforms in minimally invasive surgery [6]. Traditional training models based on Halsted’s “see one, do one, teach one” must be considered obsolete nowadays, due to patient safety requirements as well as current working time restrictions and the ever-changing demand for work-life-balance [7, 8]. In a recent survey from our working group, Swiss teaching hospitals mandate a structured laparoscopic curriculum only in 15% of all centers, although most department chairs support integration of simulation and performance-based assessment into residency training. In Switzerland, general surgical residency is a 6-year postgraduate training program regulated by the Swiss Institute for Medical Education (SIWF). The first 2 years typically focus on basic surgical training, followed by progressive operative responsibility during the subsequent years. Although simulation-based training is encouraged, its integration into structured curricula remains heterogeneous across institutions. This variability underscores the need for standardized, proficiency-based simulation curricula early in surgical training. The efficiency of virtual reality simulation training (VRST) in learning to perform a Lap-C has been evaluated in multiple studies, summarized in several meta-analyses [9–11]. However, a significant methodological heterogeneity was noted with difficulties in recent studies evaluating VRST. One recent study out of the “*Innosuisse Proficiency”* project (www.surgicalproficiency.ch) demonstrated that training per se improved laparoscopic skills, regardless of the simulator or sequence of exercises, while a subsequent work found that even intensive training amended with adjuncts as mental imagery does not necessarily translate into superior clinical practice [12, 13]. These findings underscore the urgent need for efficient, proficiency-based training formats [14], especially VRST curricula that specifically address necessary skills and procedural proficiency in order to adopt knowledge and abilities to perform Lap-C. Therefore, we developed a novel, structured training curriculum addressing the various skills needed to proficiently perform Lap-C. The present study aimed to evaluate the effect of a structured VR simulation curriculum compared with deliberate practice on laparoscopic performance in a standardized porcine cholecystectomy model. We hypothesized that residents undergoing this intensive structured training curriculum would demonstrate superior performance, assessed by blinded observers using the Global Operative Assessment of Laparoscopic Skills (GOALS) score [15].

## Materials and methods

This study was conducted as part of the 41st annual Davos Course in 2024, an international surgical training course in Davos, Switzerland (www.davoscourse.ch). This six-day course offers a blended learning experience with theoretical parts and a strong emphasis on hands-on training (open, laparoscopic & robotic).

All trainings were carried out using LaparoS® VR trainers with the most recent software (VirtaMed AG, Schlieren, Switzerland). The company provided the simulators without any financial benefit and without any direct sponsoring. Employees of the companies were present during the training and experienced laparoscopic surgeons were instructors. The study protocol was written independently and only approved before recruitment. VirtaMed had no influence on data collection, statistical analysis nor drafting of the manuscript.

The structured VR simulation curriculum was developed within the Swiss “Proficiency” research initiative (www.surgicalproficiency.ch) and consisted of 23 predefined exercises performed over three consecutive training days. The curriculum was organized into four modules: (1) basic psychomotor skills including camera navigation, depth perception, and instrument coordination; (2) bimanual dexterity and efficiency tasks; (3) procedure-related dissection tasks simulating critical steps of laparoscopic cholecystectomy; and (4) a complete simulated laparoscopic cholecystectomy run-through. The curriculum focused mainly on techniques necessary to safely dissect the hepatocystic triangle, as recommended by recent guidelines [16].

Progression within the curriculum required completion of predefined performance benchmarks before advancing to subsequent tasks. A detailed list of exercises is provided in appendix 1.

All study participants gave written informed consent to participate in the study. The Swiss Ethics Committee (Swissethics.ch) grants a general waiver for the use of purely anonymized data, and therefore no ethics votum was obtained (Swiss Federal Act on “Research involving Human Beings 810.30”). All methods were carried out in accordance with relevant guidelines and regulations.

Study participants were eligible to participate in this study, if they were enrolled in the basic module of the Davos Course and gave their written consent to participate. Criteria to be enrolled in the basic module are clinical experience of less than 3 years as a surgical trainee/resident, and less than 20 appendectomies or 10 cholecystectomies, respectively. All participants are surgical trainees enrolled in general surgery training programs.

For each participant, we collected the following data: Demographics and previous surgical experience using a questionnaire.

**Fig. 1:**
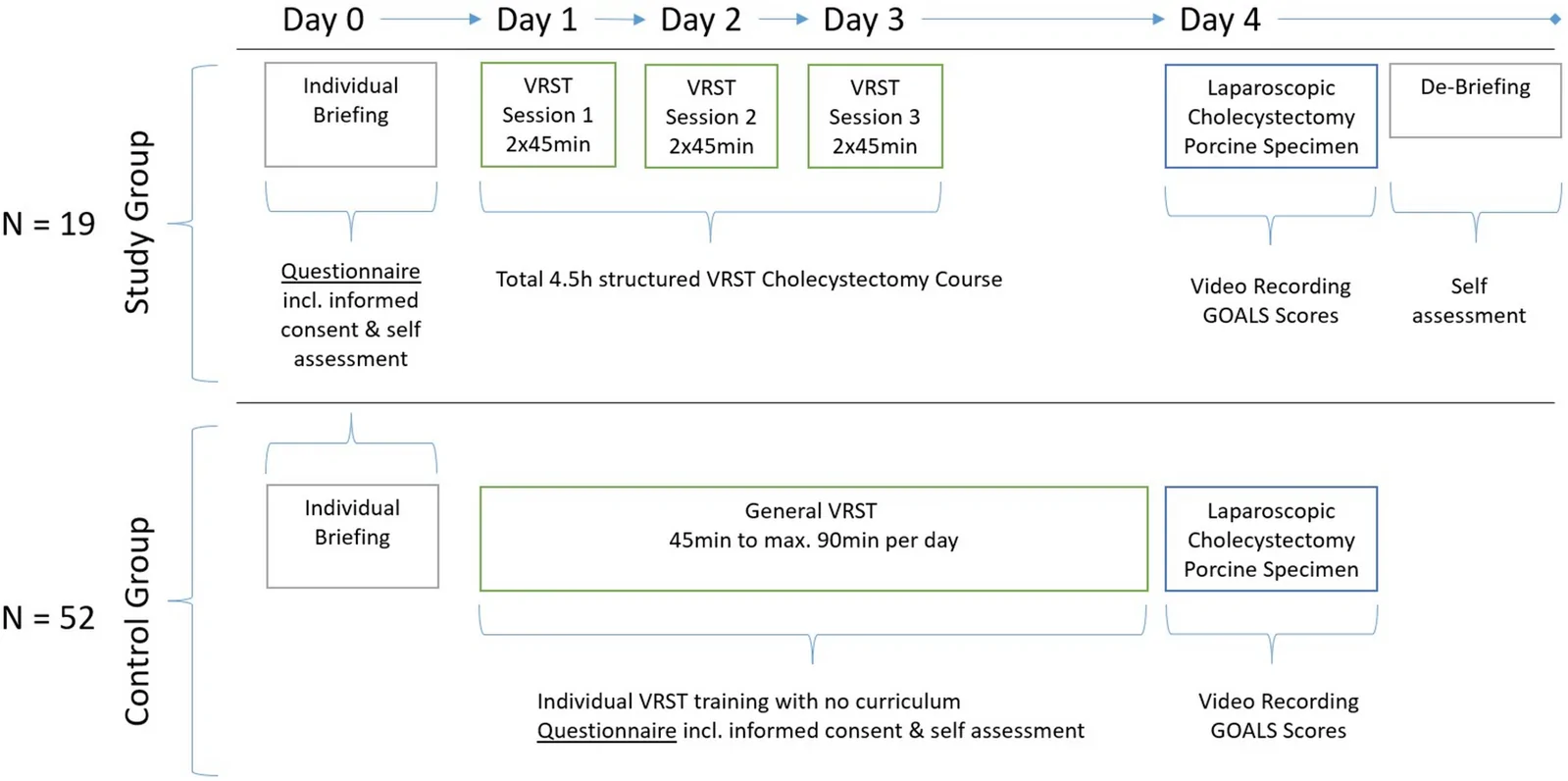
Study design

Prior to the course, participants in the basic course were offered a spot in the structured VRST curriculum via E-Mail and could book it in advance through an online form. Participants were assigned to the structured VRST curriculum in the order of their response. All remaining participants were subject to deliberate practice VRST during their course, where they were assigned to individual time slots of at least 45 min per participant, but no more than 90 min per day. Participants in the intervention group were given a three-day schedule with 90 min of training (2 × 45 min) per day with a short break in between the individual training blocks (Fig. 1). Each participant was given a brief walkthrough of the simulator to familiarize them with the system.

At the end of the course, all participants performed a laparoscopic cholecystectomy on a conventional box trainer containing a porcine liver specimen using different commercially available mobile laparoscopy units (Storz TelePack, Karl Storz SE & Co. KG, Tuttlingen, Germany), of which we recorded videos of the full procedure. Cholecystectomy performance was assessed using the GOALS score [15]. All raters are board-certified surgeons with extensive experience in laparoscopic surgery. All raters were familiar with the GOALS instrument and received a standardized briefing regarding scoring criteria prior to assessment. Raters were blinded to group allocation and participant identity. As an amendment to the GOALS score, laparoscopic performance was subjectively assessed by assigning a numerical rating score (NRS) between 0 and 10.

### Statistical analyses

Statistical analyses were performed using a recent version of the R statistical software (www.r-project.org). A two-sided *p* value of less than 0.05 was considered statistically significant. Continuous data were expressed as median values with 25 and 75% percentiles and compared by Mann–Whitney-*U*-Test. Categorical variables were compared using Chi^2^-statistics.

To assess the consistency of expert evaluations across multiple raters, we computed the Intraclass Correlation Coefficient (ICC) using a two-way random-effects model with average measures and absolute agreement [ICC(2,k)]. This approach is appropriate when both raters and subjects are considered representative of a broader population and when agreement (rather than mere consistency) is the focus. ICC values are interpreted according to common benchmarks: values below 0.50 are considered poor, 0.50–0.75 moderate, 0.75–0.90 good, and > 0.90 excellent reliability.

## Results

Eighty-six participants were enrolled in the basic module of the Davos Course, of whom 71 (82.5%) could be analyzed. Due to technical reasons, six videos could not be assessed, and nine participants in the control group refused to participate.

### Baseline data

Participants did not differ significantly in their baseline data. Previous laparoscopic experience in appendectomies and cholecystectomies also showed no statistically significant differences, with only two participants in the control group having exceeded 20 cases and one participant in the intervention group with more than 20 appendectomies done (Table 1).

**Table 1:** Baseline data for control and intervention group

	Control (*n*, %)	Intervention (*n*, %)	*p*-value
Year of birth (median (IQR))	1995 (3)	1995 (3)	*p* = 0.281
Gender			
Female	31 (59.6%)	13 (68.4%)	*p* = 0.499
Male	21 (40.4%)	6 (31.6%)	
Handedness			
Right-handed	43 (91.5%)	18 (94.7%)	*p* = 0.652
Left-handed	4 (8.5%)	1 (5.3%)	
Years of surgical training (median (IQR))	2 (1)	2 (1)	*p* = 0.762
Previous laparoscopic appendectomies			
> 20	0 (0%)	1 (5.3%)	*p* = 0.64
≤ 20	47 (100%)	18 (94.7%)	
Previous laparoscopic cholecystectomies			
> 20	2 (4.3%)	0 (0%)	*p* = 0.90
≤ 20	50 (95.7%)	19 (100%)	

### Goals scores

**Fig. 2:**
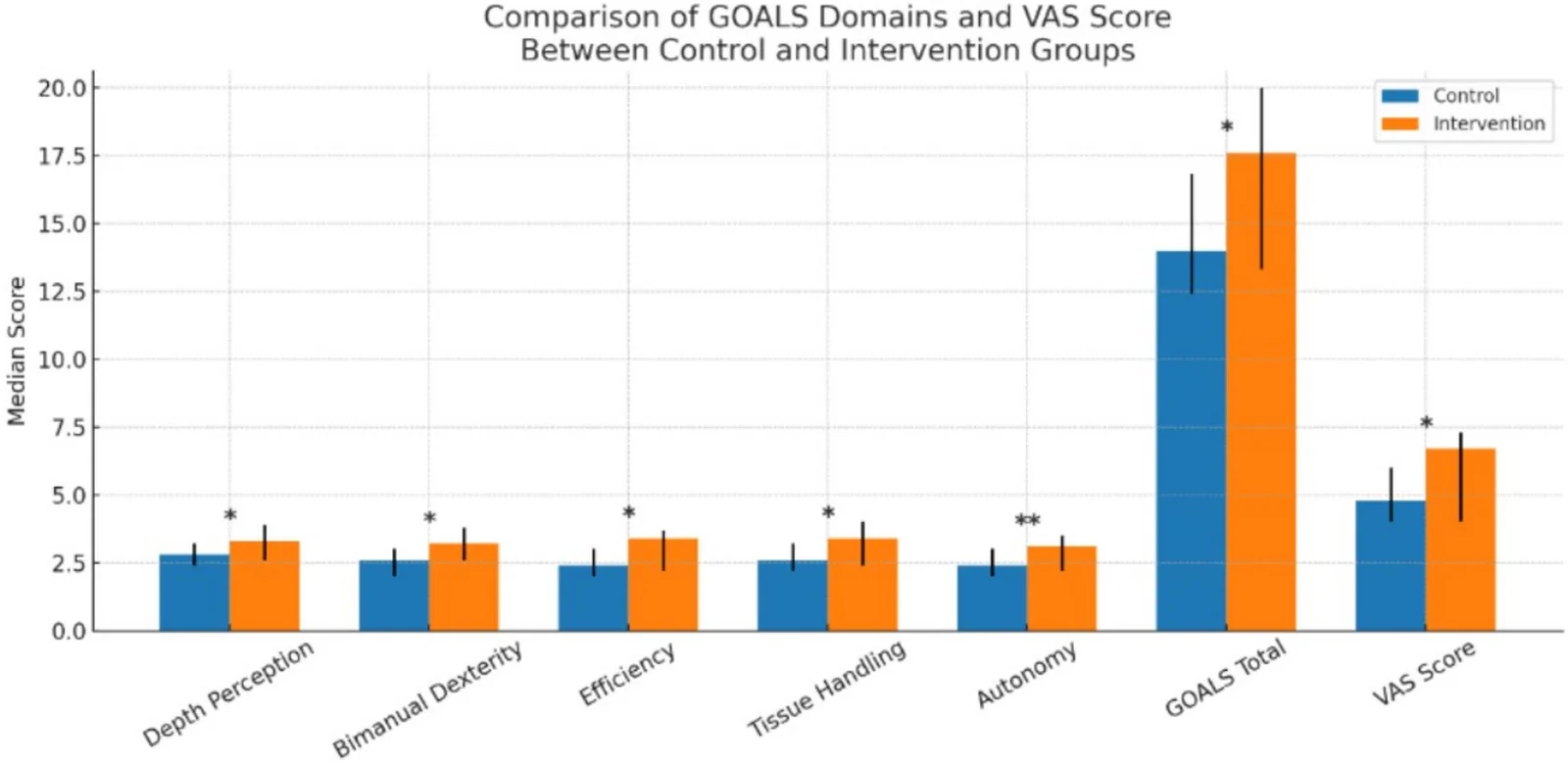
Comparison of median values of the GOALS domains and NRS score between Control and Intervention Groups. *GOALS* Global Operative Assessment of Laparoscopic Skills; Asterisks mark statistically significant differences (**p =  < 0.01, *p =  < 0.05)

Participants who underwent the structured simulator-based training demonstrated significantly better performance than those in the control group across all assessed domains of the GOALS as well as in the sum-score (Fig. 2). Specifically, the intervention group scored higher in ‘depth perception’ (U = 397.5; *p* = 0.019), ‘bimanual dexterity’ (U = 387.5; *p* = 0.014), ‘efficiency’ (U = 375.5; *p* = 0.010), ‘tissue handling’ (U = 411.0; *p* = 0.029), and ‘autonomy’ (U = 367.5; *p* = 0.008). The total GOALS score was also significantly higher in the intervention group (Median = 17.6 points) compared to the control group (Median = 14.0 points; U = 382.5, *p* = 0.013). Furthermore, subjective evaluations using the NRS revealed significantly better ratings for the intervention group (Median = 6.7) versus control (Median = 4.8; U = 419.0, *p* = 0.036) (Table 2).

**Table 2:** Median (25%, 75% percentiles) for control and intervention group for GOALS total and its domains

GOALS	Control	Intervention	Mann–Whitney U	*p* value
Depth perception	2.8 (2.4, 3.2)	3.3 (2.6, 3.9)	397.5	0.019
Bimanual dexterity	2.6 (2, 3)	3.2 (2.6, 3.8)	387.5	0.014
Efficiency	2.4 (2, 3)	3.4 (2.2, 3.7)	375.5	0.010
Tissue handling	2.6 (2.2, 3.2)	3.4 (2.4, 4)	411	0.029
Autonomy	2.4 (2, 3)	3.1 (2.2, 3.5)	367.5	0.008
GOALS total	14 (12.4, 16.8)	17.6 (13.3, 20)	382.5	0.013
NRS score	4.8 (4, 6)	6.7 (4, 7.3)	419	0.036

*GOALS* Global Operative Assessment of Laparoscopic Skills, *NRS* numerical rating scale

### Inter-rater correlation

The inter-rater agreement for the overall GOALS score was good with an ICC of 0.795 (95% CI 0.732 – 0.850, *p* < 0.001). For the subscales of the GOALS score, the inter-rater agreement was moderate albeit statistically significant in all items (Table 3).

**Table 3:** Inter-observer reliability of the five raters’ GOALS Scores and VAS score using ICC (2,k)

GOALS	ICC (2,k)	95% CI	*p*-value	Interpretation
Depth perception	0.601	[0.507, 0.694]	< 0.001	Moderate
Bimanual dexterity	0.677	[0.592, 0.757]	< 0.001	Moderate
Efficiency	0.612	[0.518, 0.703]	< 0.001	Moderate
Tissue handling	0.646	[0.557, 0.732]	< 0.001	Moderate
Autonomy	0.665	[0.579, 0.747]	< 0.001	Moderate
GOALS total	0.795	[0.732, 0.850]	< 0.001	Good
NRS	0.74	[0.665, 0.807]	< 0.001	Good-Moderate

ICC > 0.90 = Excellent, ICC 0.75–0.90 = Good, ICC < 0.75 = Mode

## Discussion

In this study, we assessed the effect of a newly developed, structured VRST curriculum on laparoscopic performance of surgical novices. Participants were offered participation in a three-day structured VRST training tackling basic exercises as well as key steps of a safe cholecystectomy. Controls were assigned to standard deliberate practice on the same VR simulation trainer. Laparoscopic performance was assessed in a well-established porcine cholecystectomy trainer by surgeons with different levels of experience using the GOALS tool.

Participants in the intervention group performed significantly better in the overall and all subscales of the GOALS Score as well as in the NRS.

VRST is long known to improve surgical performance in acquiring laparoscopic skills as well as other complex psychomotor skills, e.g., gastrointestinal endoscopy [17], endovascular surgery [18] or knee arthroscopy [19]. The first studies showing that VRST improves performance in laparoscopic cholecystectomy have been published more than 20 years ago and paved the way for a broader development of VRST training curricula [20, 21]. However, demonstrating meaningful gains in junior trainees has been notoriously difficult. A systematic review and meta-analysis of Humm et al. showed that VRST reduced operative time and retrieved higher OSATS (Objective structured assessment of technical skills) scores in trainees but found no statistically significant difference in GOALS scores [9]. However, in most of the studies assessed, there were different didactic elements amended to VRST, including case-based learning, box-training and OR participation [22, 23]. Therefore, it is difficult to compare the different interventions and their outcomes. Moreover, most studies in the field include only small numbers of participants. Hogle et al. compared 13 first-year residents and 21 general surgical interns for their study, that failed to prove VRST superior to no additional training [24]. Another study by Våpenstad et al. also could not demonstrate a benefit for VRST compared to controls, arguing that missing haptic feedback could be responsible for the lack of skill-transfer [25]. When analyzing studies reporting GOALS, Humm et al. could not demonstrate a benefit for VRST over regular training but did so when analyzing studies reporting OSATS scores [9].

In the present study, we evaluate 72 participants, rendering it one of the largest prospective studies to date in this field. Moreover, our intervention did not encompass any other than the VRST curriculum, reducing potential bias.

On the other hand, the study has several limitations: Due to organizational restraints we could not include more than 20 participants in the intervention group, of whom 19 were assessed in the final analysis. Allocation to the structured curriculum was not randomized but based on voluntary registration and response order. Therefore, self-selection bias cannot be excluded. It is possible that participants opting for the structured curriculum were more motivated or particularly interested in simulation-based training, which may have influenced performance outcomes.

However, intervention and control group did not show any differences in previous experience or other relevant baseline data. Despite the use of a porcine cholecystectomy model instead of e.g., real operations under clinical conditions, it is a well-established means of assessing laparoscopic performance that has been used in various previous studies [21, 26].

Total simulator exposure time in the control group was not systematically recorded. Consequently, it cannot be excluded that differences in cumulative simulator time may have partially contributed to the observed performance differences between groups. Future studies should control for total training duration to better isolate the independent effect of structured curricula.

Ultimately, this is to date one of the largest cohort studies assessing the effectiveness of a dedicated VRST curriculum on laparoscopic performance and the first to evaluate the effectiveness of a VRST curriculum in a novel simulator model.

In Switzerland, the Surgical Proficiency Initiative (www.surgicalproficiency.ch) aims to integrate structured VR simulation training into national surgical residency curricula by providing standardized modules, proficiency benchmarks, and faculty support. Our findings contribute directly to this initiative by demonstrating that structured VRST curricula improve early laparoscopic performance, supporting their potential role in harmonized technical skill development across training centers.

## Conclusion

In this prospective comparative study, a structured VR simulation curriculum improved laparoscopic performance in a standardized porcine cholecystectomy model compared with unstructured deliberate practice. As GOALS reflects global laparoscopic operative performance rather than procedure-specific competency, the findings should be interpreted as improved overall laparoscopic performance within a cholecystectomy context. These findings support the integration of structured, proficiency-based VR simulation curricula into early surgical training to enhance technical skill acquisition and procedural competence. Further studies are required to determine transferability to clinical operating room performance.

## Supplementary Information

Below is the link to the electronic supplementary material.

Supplementary file1 (PDF 634 KB)
