# Supplementary material for: Effect of a structured virtual reality simulation curriculum on laparoscopic performance in a porcine cholecystectomy model: a prospective comparative study
Source: Surg Endosc. 2026 Apr 1;40(6):4839–45. doi: 10.1007/s00464-026-12755-z (PMC13687328; doi:10.1007/s00464-026-12755-z)
Supplement: Supplementary file 1 [file 464_2026_12755_MOESM1_ESM.pdf]

Cholesystectomy Davos 2024

LaparoS™ Essential Skills | LaparoS™ General Surgery

23 scenes in 5 sections

Description	Cholecystectomy course consisting of 3x90 min sessions.<br>Day 1 - Clipping and cutting<br>Day 2 - Resection of the gall bladder<br>Day 3 - Dissection of the hepatocytic triangle<br><br>When the excersises for a day are completed and there is time left, return to repeat excersises from that day.
Objective	Improve manual laparoscopic skills and train towards performing a cholecystetomy .

Introduction

3 ★

Practice

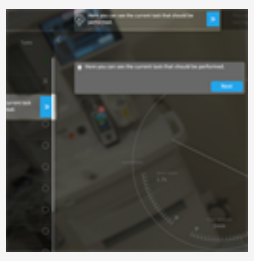

Guided - System Introduction

Video Tutorials

75%

1

Day 1 - Clipping and cutting

30 ★

Practice

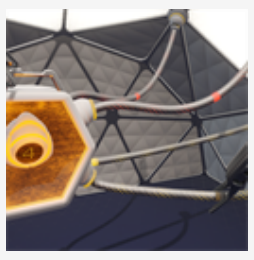

Clipping

Clipping and ligation

70%

3

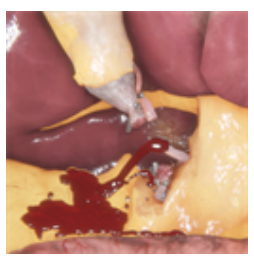

Guided - Vascular Injury

Complication Handling

70%

1

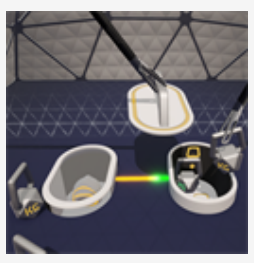

Collect the weights

Bimanual coordination

70%

3

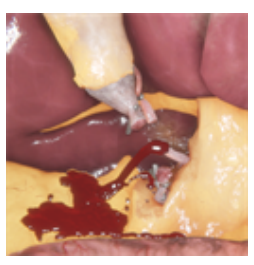

Unguided - Vascular Injury

Complication Handling

70%

3

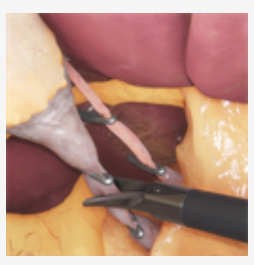

Guided - Cystic Pedicle Dissection

Clipping and Cutting

70%

1

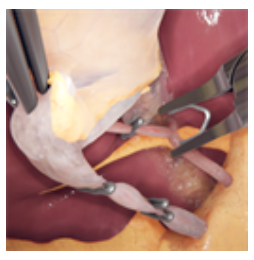

Unguided - Cystic Pedicle Dissection: Variation III
Clipping and Cutting
70%
3

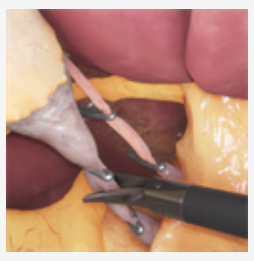

Unguided - Cystic Pedicle Dissection
Clipping and Cutting
70%
3

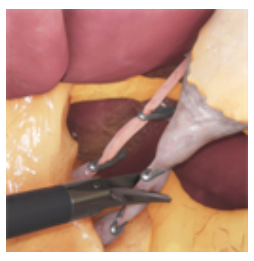

Unguided - Cystic Pedicle Dissection (Situs Inversus)
Clipping and Cutting
70%
1

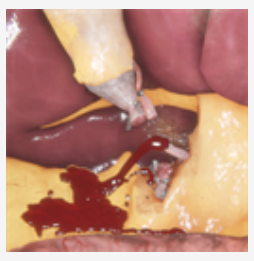

Unguided - Vascular Injury
Complication Handling
70%
3

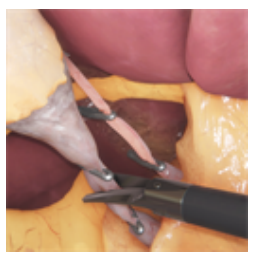

Unguided - Cystic Pedicle Dissection
Clipping and Cutting
70%
3

Day 2 - Gall bladder resection

18

Practice

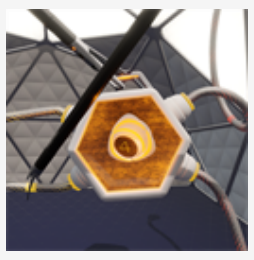

Cutting - Crossing instruments
Cutting
70%
3

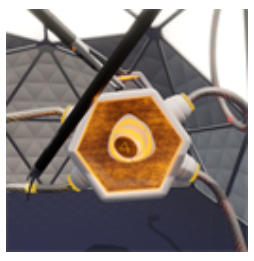

Cutting - Crossing instruments
Cutting
70%
3

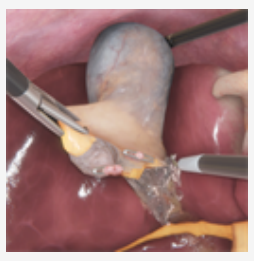

Guided - Gallbladder Resection
Resection
70%
1

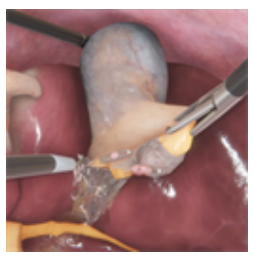

Guided - Gallbladder Resection (Situs Inversus)
Resection
70%
1

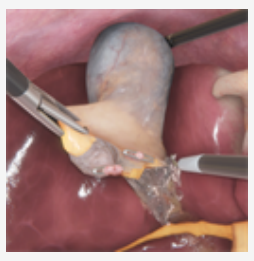

Unguided - Gallbladder Resection
Resection
70%
3

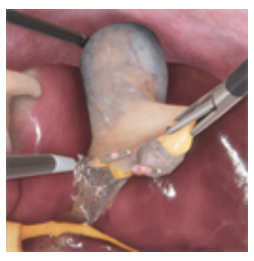

Unguided - Gallbladder Resection (Situs Inversus)
Resection
70%
3

Day 3- Dissection of the hepatocytic triangle

15 ★

Practice

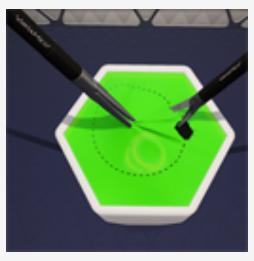

Circular cutting - Difficult

Cutting

70%

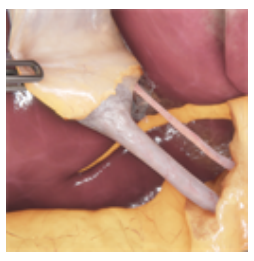

Guided - Hepatocystic Triangle

Exposure of Structures

70%

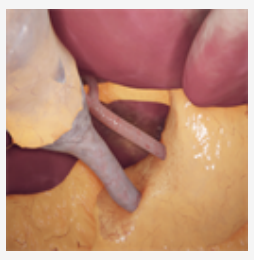

Unguided - Hepatocystic Triangle: Variation III

Exposure of Structures

70%

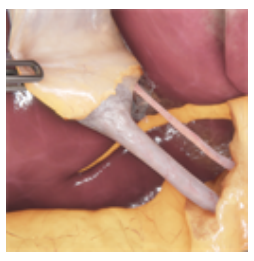

Unguided - Hepatocystic Triangle

Exposure of Structures

70%

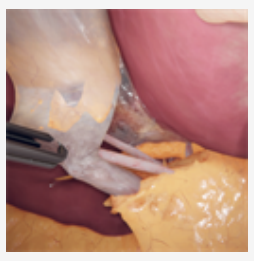

Unguided - Hepatocystic Triangle: Variation IV

Exposure of Structures

70%

Complete procedure

3 ★

Practice

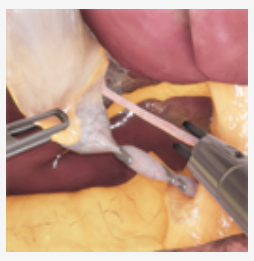

Unguided - Cholecystitis

Free Training

70%
